# Supplementary material for: Development of a computer-aided design software for the quantitative evaluation of aesthetic damage
Source: PLoS One. 2019 Dec 18;14(12):e0226322. doi: 10.1371/journal.pone.0226322 (PMC6919621; doi:10.1371/journal.pone.0226322)
Supplement: S1 Code site — 1. (ZIP) [file pone.0226322.s001.zip › [EESC_jr][Projeto]Relatorio_07_21/vendors/select2/docs/_includes/options/compatibility.html]

# Backwards compatibility

{% include options/compatibility/matcher.html %}
{% include options/compatibility/initial-selection.html %}
{% include options/compatibility/query-function.html %}
{% include options/compatibility/text-input.html %}
